# Evaluation of Diffuse Reflectance Spectroscopy Vegetal Phantoms for Human Pigmented Skin Lesions

**DOI:** 10.3390/s24217010

**Published:** 2024-10-31

**Authors:** Sonia Buendia-Aviles, Margarita Cunill-Rodríguez, José A. Delgado-Atencio, Enrique González-Gutiérrez, José L. Arce-Diego, Félix Fanjul-Vélez

**Affiliations:** 1 Biomedical Engineering Group, TEISA Department, Universidad de Cantabria, 39005 Santander, Spain; sonia.buendia@alumnos.unican.es (S.B.-A.); arcedj@unican.es (J.L.A.-D.); 2 Biomedical Optics Group, Polytechnic University of Tulancingo, Tulancingo 43629, Mexico; margarita.cunill@upt.edu.mx (M.C.-R.); jose.delgado@upt.edu.mx (J.A.D.-A.); enrique.gonzalez@upt.edu.mx (E.G.-G.)

**Keywords:** diffuse reflectance spectroscopy, pigmented skin lesions, biological tissue phantoms, skin diagnosis, principal component analysis

## Abstract

Pigmented skin lesions have increased considerably worldwide in the last years, with melanoma being responsible for 75% of deaths and low survival rates. The development and refining of more efficient non-invasive optical techniques such as diffuse reflectance spectroscopy (DRS) is crucial for the diagnosis of melanoma skin cancer. The development of novel diagnostic approaches requires a sufficient number of test samples. Hence, the similarities between banana brown spots (BBSs) and human skin pigmented lesions (HSPLs) could be exploited by employing the former as an optical phantom for validating these techniques. This work analyses the potential similarity of BBSs to HSPLs of volunteers with different skin phototypes by means of several characteristics, such as symmetry, color RGB tonality, and principal component analysis (PCA) of spectra. The findings demonstrate a notable resemblance between the attributes concerning spectrum, area, and color of HSPLs and BBSs at specific ripening stages. Furthermore, the spectral similarity is increased when a fiber-optic probe with a shorter distance (240 µm) between the source fiber and the detector fiber is utilized, in comparison to a probe with a greater distance (2500 µm) for this parameter. A Monte Carlo simulation of sampling volume was used to clarify spectral similarities.

## 1. Introduction

In recent years, the number of reported cases of cutaneous neoplasms has increased considerably worldwide [1]. Among these neoplasms is melanoma, which is derived mainly from the abnormal production of atypical melanocytes and characterized by a high degree of metastasis [2,3]. In Mexico, melanoma ranks third in skin cancer incidence, accounting for 7.9% of skin cancers, and is the cause of 75% of deaths from this type of neoplasm [4,5]. As reported by the American Cancer Society, the survival rate of a person with melanoma will largely depend on how far the cancer has spread at the time of diagnosis [6]. Unfortunately, approximately 4% of cases are detected when the cancer has spread to other parts of the body, reducing the survival rate to 35% [6].

Currently, dermatologists employ several methods and techniques for differentiation and detection between melanoma and benign pigmented skin lesions, that usually lead to invasive, painful, costly, and delayed procedures, such as biopsy. For these reasons, the development of more efficient tools has been encouraged, particularly the use of non-invasive techniques based on optics, for the preventive diagnosis of suspicious skin lesions. Several optical techniques have been used to analyze and differentiate human skin tissues, especially for the distinction between healthy tissue and cutaneous melanoma, including NIR-SWIR spectroscopy, in vivo confocal microscopy, optical coherence tomography (OCT), and multispectral image analysis, which allow one to obtain detailed information on the composition and structure of the skin. In particular, NIR-SWIR spectroscopy in the 1000–2000 nm range has shown greater sensitivity in identification of elements such as collagen, lipids, and water compared to the visible and NIR range. In this work, diffuse reflectance spectroscopy (DRS) was used because it is a relatively fast technique with the ability to provide detailed information on tissue composition. Although DRS is sensitive to external conditions such as illumination and angle of incidence, it remains a valuable technique whose implementation and effectiveness has been proven in the characterization of human skin. Although more advanced techniques such as OCT exist, they are more expensive and complex. DRS stands out for being an affordable, fast, and noninvasive technique, with a wide range of spectral analysis, which was decisive for our choice. Through multivariate analysis, such as PCA, we were able to extract key patterns from the visible spectra, facilitating the identification of changes in tissue composition. This makes DRS ideal for the diagnosis and monitoring of skin lesions, by offering valuable data on chromophores such as melanin and hemoglobin, providing valuable data on the physiology of the lesions.

Diffuse reflectance spectroscopy has been used for the diagnosis of non-melanoma skin cancer [7,8], including the use of physiological models [8,9]. This technique is characterized by being easy to implement. Other clinical applications of DRS have been reported, such as the determination of the optical properties of the skin [10,11], blood flow measurement [12,13], detection of skin and breast cancer [7,8,14,15,16], and the discrimination between healthy and diseased tissues, among others [17,18]. The above approaches require a significant number of samples to be able to adjust the parameters of the technique.

Various investigations have determined that the enzyme tyrosinase plays an important role in the formation of melanin present in pigmented lesions such as melanoma [19,20,21,22]. Lin et al. demonstrated the presence and distribution of this enzyme in biopsies of melanomas in stages II and III by scanning electrochemical microscopy (SMSE) [23]. Similarly, tyrosinase was also observed in the brown spots that appear on the skin of banana fruit [24]. These similarities between human skin pigmented lesions and brown spots on banana fruit could potentially be exploited for the development of diagnostic equipment. In particular, brown spots on banana fruit could serve as optical phantoms for human skin pigmented lesions. This would allow the use of very cheap and affordable non-animal samples in the tests of new dermatological equipment based on optical techniques. In addition, in some cases, depending on the nature of the optical diagnostic devices, it would prevent the thorough documentation process required in the informed consent studies in humans at the first stages.

Previous studies on the detection and differentiation between melanoma and benign pigmented skin lesions have shown that the differences between the two types of lesions lie mainly in cellular, histological, and molecular characteristics. However, due to the limitation of not having spectral signatures of melanomas in this work, our focus was on the spectral and visual differentiation between pigmented and non-pigmented skin, with a particular emphasis on the similarity of nevi and spots.

In this work, brown spots on the skin of banana fruit formed by the ripening process are analyzed as a possible biological tissue phantom of the pigmented lesions on human skin. The similarity they present with nevi or moles in terms of symmetry and color tonality is a potential indicator of similar optical properties, that could be exploited by DRS. To this aim, the characteristic spectral curves of nevi of volunteers with different skin phototypes were recorded and compared with the spectral curves of brown spots on banana skin. All of the curves were obtained using DRS for two groups of volunteers and spots using two distinct probes: for the initial set of spectra, a homemade probe with 980 µm diameter fibers was utilized; while for the subsequent set of spectra, a commercial probe with two 200 µm diameter fibers was employed. A digital image processing algorithm was implemented for the analysis of the areas of interest, which allowed the determination of the dimensions of each lesion or spot, as well as the calculation of the average value of the color intensity in the three channels of the RGB system for comparison purposes. Comparative analyses of the spectral characteristics of both pigmented skin lesions and brown banana spots were performed, as well as the evaluation of their color properties. In addition, advanced spectral characteristics were implemented and compared using principal component analysis (PCA). Finally, a Monte Carlo simulation of the sampling volume was conducted for the fiber-optic probes used to reinforce their suitability and confirm their relevance to the study, thereby providing support for the obtained results. To our knowledge, and based on an exhaustive search of published research related to our investigation, no similar studies have been reported on the comparison of human and banana skin performed with DRS. The results indicate significant similarity between the spectral, area and color characteristics of both human skin pigmented lesions and brown banana spots, particularly at specific ripening stages.

## 2. Materials and Methods

### 2.1. Selection of Brown Spots on Banana Skin

In this study, 48 banana spots from different samples at different ripening stages were selected and spectrally evaluated. In addition, their visual characteristics, such as diameter and color, were analyzed using photographic images. A banana sample was selected weekly from a bunch belonging to the musa paradisiaca variant (Musa Cavendish Colla). Specifically, bananas belonged to the local variant called Tabasco banana, the easiest commercial variant to be acquired throughout the year. The selected samples had lengths between 18 and 24 cm, and approximate diameters of 3.5 cm. The diffuse reflectance spectral measurements were performed using a “homemade probe” of two 980 µm diameter plastic optical fibers, with a separation of 2500 µm between the core of the source fiber and the detector fiber. A detailed explanation of the elements comprising the probe and its geometrical setting is provided in Section 2.3. It is important to note that this probe was designed for previous research to facilitate the use of diffusion theory formulae in optical property recovery tasks, given the optimal source–detector distance.

**Figure 1:**
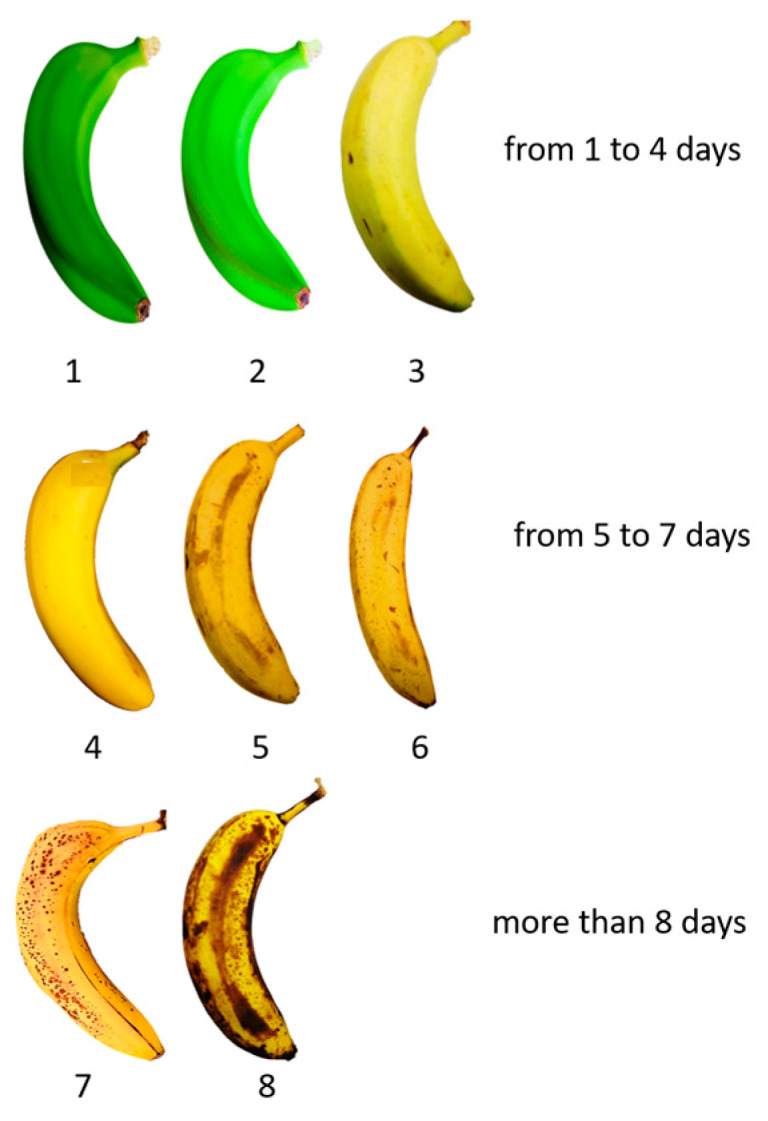
Scale (1–8) for the classification of the different ripening stages of bananas, associated with changes in the color of the peel, following the one proposed by Escalante et al. [25].

Initially the samples presented a firm state with a predominantly green coloration, characteristic of ripening stages 1 and 2, according to the stages of ripening based on the changes in skin tone of banana fruit [25]. Following this classification, we identified 8 stages for the samples of this study as follows: stage 1, dark green; stage 2, green; stage 3, green–yellow, more green than yellow, some yellow areas are visible; stage 4, more yellow than green, green areas still visible; stage 5, firm yellow skin with small brown spots; stage 6, all yellow and presence of moderately large spots; stage 7, yellow with large brown spots; stage 8, more brown than yellow and even with some areas near to decomposition. These stages are shown in Figure 1.

In addition, measurements were taken in a second group comprising 24 spots in samples of the same variant, with maturation stages between 4 and 7. It should be noted that these measurements were especially focused on spots in the initial stages of development with a low amount of brown pigmentation, which were called “dim spots”, located mainly in samples at maturation stage 5. These spectra were acquired using a different probe to the one described above, which we refer to as the “commercial probe”.

Banana grading stages usually change between 5 and 8 from a “green” to an “overripe” state, depending on various visual and sensory features [26,27,28]. In order to properly evaluate a spot, spectral measurements were carried out in an area without the presence of other spots or lesions near to the spot under measurement. In addition, measurement areas were photographically monitored under the same illumination conditions throughout the study. The average ambient temperature of the laboratory was 22 °C throughout the experimentation period.

### 2.2. Human Nevi in Volunteers

Measurements on human nevi or moles were carried out on a total of 10 volunteers with the homemade probe after informed consent; the volunteers ranged from in their twenties to their thirties. For the recording of the spectral curves, two areas were chosen for each participant: (a) nevi, which were located on the arm, forearm, back, and palm of the hand; and (b) a nearby area without the presence of other lesions, which we call healthy skin, because during the previous interrogation volunteers declared themselves to be free of skin diseases. At the same time, photographic images of the described regions were captured under controlled illumination conditions for documentation and analysis purposes during this research work. In addition, a second group of measurements was performed, for which eight new volunteers were recruited, most of whom were between eighteen and twenty years of age. Following the methodology previously described, regions similar to those already mentioned were selected for nevus measurements. However, in one of the volunteers, measurements were carried out on three nevi located on different parts of the body; a total of 10 nevi were measured, identified in this paper as N1, N2, N3, N4, N5, N6, N7, N8-1, N8-2, and N8-3.

### 2.3. Diffuse Reflectance Spectroscopy System and Measurement Procedure

**Figure 2:**
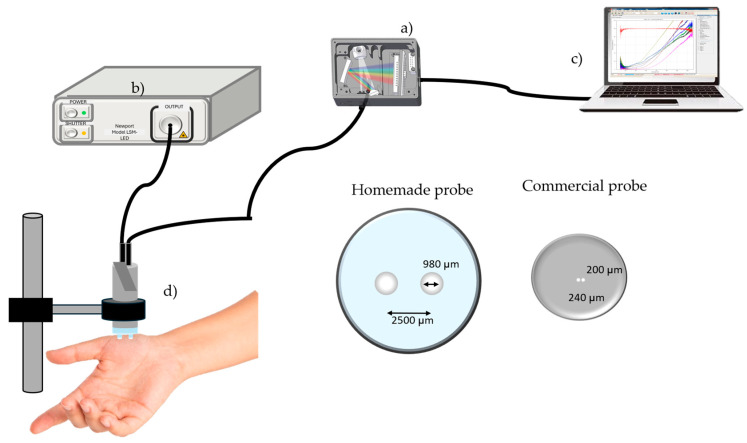
Experimental configuration implemented for the acquisition of diffuse reflectance spectra for banana tissue and volunteers participating in this study: (**a**) Mini spectrometer; (**b**) tungsten halogen light source; (**c**) personal computer equipment for spectral analysis; (**d**) bifurcated fiber-optic probe and zoom of the geometries of the optical probes used.

A DRS system was implemented for the acquisition of spectral curves in both tissue types, as illustrated in Figure 2. The configuration includes a class I light source (LSM-T-S Tungsten-Halogen Mini-Light Source, Newport Corporation, Irvine, CA, USA) emitting in a spectral range of 300–1700 nm. The source is connected to a bifurcated fiber-optic probe via SMA connectors. It is a tungsten halogen source with a power consumption of 12 W and an optical emission of around 10 mW for the whole spectrum. Final irradiation on the sample, taking into account fiber loss, is similar to that typically encountered in biomedical optics applications (hundreds of mW per square centimeter). The source achieves thermal stability in less than 5 min and operation is optimal over a temperature range of 5 to 35 °C. The other bifurcated end of the probe is coupled to a spectrometer (USB 4000, Ocean Insight, Orlando, FL, USA). The homemade probe was designed using a plastic optical fiber (Model 02-356, Edmund Optics, York, UK), and consists of source-fiber and a detector-fiber paths, with core diameters of 980 µm. The fibers are placed parallel to each other, separated by a distance of 2500 µm from center to center, and encapsulated in a transparent container of 6 mm diameter, as shown in Figure 2. A cylindrical volume of Teflon (6 cm in diameter and 3.5 cm in height) was employed as a diffuse reflectance calibration standard for the purposes of measurement. Initially, a dark reference spectrum was captured with the source shutter closed and stored for later use. The light reference spectrum was then recorded using a reflectance standard to establish 100% reflectance. All reflectance measurements were performed in a controlled dark environment to avoid spectral contamination and errors generated by other light sources. Subsequently, spots and nevi of interest were measured, as described later in this section. Data were collected in the 178 to 890 nm range with 0.17 nm resolution and stored for subsequent processing under the following conditions: the source fiber was placed at the center of the skin lesions or banana spots, in contact and without exerting pressure on them, while the detector fiber was placed on the skin or non-pigmented spots of volunteers or bananas, respectively. This process was repeated five times for statistical purposes. Average curves were calculated for each pigmented skin lesion and banana brown spot for the purpose of comparative analysis of different aspects of this study, allowing for a more comprehensive evaluation of the data. In order to test other experimental conditions, another spectrometer (BRC115P-UV-VIS/NIR, B&W Tek, Plainsboro, NJ, USA), together with the use of a second fiber-optic probe, which we refer to as the “commercial probe” (BIF200-MIXED, Ocean Insight, Orlando, FL, USA), enabled the evaluation of the similarity of diffuse reflectance spectra between banana spots and skin lesions in a second group of volunteers and bananas, as previously described (Section 2.1 and Section 2.2). This probe differs from the homemade probe in having smaller values for the distance between the center of its cores and the diameter of its cores, 240 µm and 200 µm, respectively. In addition, the core of the commercial fiber probe is made of quartz unlike the homemade probe, which, as mentioned above, is made of plastic. The second set of measurements was performed under the same conditions described above but, in particular, both fibers of the commercial optical probe were completely contained within the dimensions of both types of pigmented lesions of the second group of volunteers and bananas studied. The spectral data for this configuration were recorded for the spectral range 314–1063 nm with 0.29 nm resolution; however, for comparative purposes only the data in the 400–750 nm region were considered. This spectral range was selected because it covers most of the visible spectrum, where the main reflectance characteristics of the samples studied are found. In particular, the main optical properties of absorbers of human skin (hemoglobin and melanin) and banana skin (chlorophyll and melanin) are shown in this range. These absorption properties, together with the scattering properties of both media, provide the spectral behavior of the measured diffuse reflectance spectra. Furthermore, for wavelengths below 450 nm and above 750 nm the noise of the reflectance signal is higher.

Finally, it is important to note that in this study the homemade optical probe tip was configured with a relatively large separation between the source fiber and the detector fiber, similar to that reported in the specialized literature of biomedical optics [29,30,31]. This configuration considers the possible influence of healthy skin and banana skin without brown spots on the spectral similarity. It also allows us to use the Farrel model [32] to extract the optical properties if necessary. The choice of this probe geometry has an impact on the main research question of this paper, regarding the diffuse reflectance spectrum similarity of banana brown spots and skin nevi. In order to explore this influence, a second fiber-optic probe was studied, both theoretically and experimentally, to partially address this issue. It has been widely reported in the related literature that the tissue sampling volume of a fiber-optic probe depends on the optical properties of the turbid medium, tissue structure, and the geometry configuration of the probe [33,34]. The so-called banana shape of the sampling volume has been reported, which interrogates the tissue more deeply as the source–detector distance is increased. In Section 4 we return to this topic in connection with future research that would complement the findings in this paper.

### 2.4. Calculation of the Dimensions of Nevi and Brown Spots

Macroscopic images of spots and nevi were captured by a camera with a Sony IMX035 CMOS sensor (FL3-U3-13S2C-CS Point Gray Color Camera, Edmund Optics, York, UK) of 1.3 megapixels, a resolution of 1328 × 1048 pixels, and a sensor size of 1/3″. A 16 mm focusing lens (VIS-NIR-Edmund Optics, York, UK) with an aperture of f/1.4 to f/16 was attached to the camera. Image processing was performed using the Point Gray FlyCap2 software (version 2.14.3.1). Images were captured under similar lighting conditions. In this study, a vertical setup was established. The working distance was measured from the frontal plane of the lens to the surface of the sample, either the banana or the skin of the volunteer.

A script was developed in MATLAB^®^ R2022b (MathWorks, Inc., Natick, MA, USA) that allowed images of nevi and brown spots on the banana skin to be processed. From these images, a manual selection of points on the edges was made: one on the far right and one on the left to obtain the horizontal diameter; and then, a point on the top and bottom edge was selected to calculate the vertical diameter. To determine the dimensions of the lesions, the image of a millimeter-graduated ruler was used, captured under the same lighting and focus conditions used for the nevi and spots. This image served as a reference to establish the proportionality relationship between the graduated ruler and the values obtained from the lesions. Finally, given the similarity of the shape of the lesions with an ellipse, the diameters obtained were used to calculate the approximate area of each lesion using the formula for the area of an ellipse.

The dimensions of skin lesions are a key parameter in the study of pathologies such as melanoma, where the literature places special emphasis on the size of suspicious lesions, particularly those with a diameter equal to or greater than 6 mm. In this study, although the spots on the banana skin presented smaller dimensions than those commonly reported for melanomas, the measurement of the diameter of the lesion is still an important parameter. This allows us to establish under which conditions the diffuse reflectance spectra of brown spots on banana and human skin lesions are similar. The dimensions of the spots on the banana were compared with those of benign nevi, especially for spots at stages of maturation later than stage 5.

### 2.5. Calculation of the Average Color Intensity in Spots, Nevi, and Healthy Skin

The average color intensity value of both types of lesions was calculated using a script developed in MATLAB^®^. This script allowed for processing the images of nevi and spots previously captured with a CMOS camera and manually selecting the central pixels of each lesion. In general, areas of 20 × 20 pixels were selected, except in cases where the presence of hair was visible or spots had a reduced size (diameter less than 1 mm), where the selection of smaller areas, of 10 × 10 pixels, was required to avoid inclusion of unwanted pixels. The program extracted the RGB channel values of the selected pixels and calculated the average for each channel. These average values allowed us to determine the hue and intensity of the color in the selected areas of each nevus and spot.

The performance of the algorithm for calculating the color of the region of interest was tested by selecting two similar healthy skin areas of 20 × 10 pixels from each volunteer: one at the location where the DRS measurement was performed and the other in a nearby region, where no spectral measurements were carried out. For each area, the shadow was calculated with the aforementioned code, finding very close values for each pair of areas.

The skin phototype of the volunteers was determined by applying the survey proposed by Silonie Sachveda [35], that takes into account the Fitzpatrick classification scale [36]. This classification scale is one of the most used today. It considers six skin phototypes, from the lightest to the darkest skin. The skin response to sun exposure is evaluated based on the skin phenotype, the generation of melanin, and the individual tanning habits. In this study, the Silonie Sachveda survey was applied to the participants, which includes a series of predefined questions with associated scores. Based on the total score obtained, participants were classified according to the Fitzpatrick scale to determine their skin phototypes.

**Figure 3:**
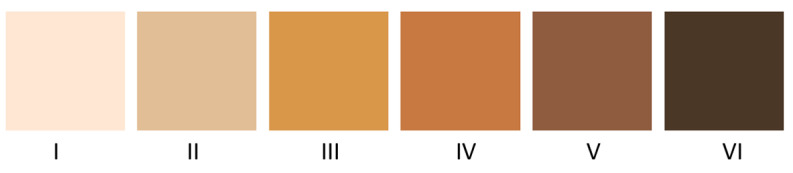
Shades of skin color ordered in ascending order according to Fitzpatrick’s classification (I–VI). The shades presented are based on those proposed by Caerwyn et al. [37].

Figure 3 shows a representation of the different skin tones, proposed by Caerwyn et al. [37] and organized according to Fitzpatrick’s classification.

**Figure 4:**
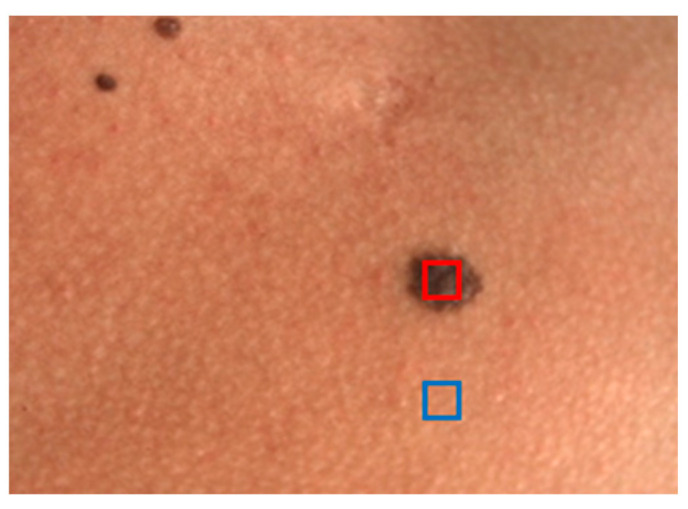
Areas selected to calculate the average color value of two of the regions of interest measured with DRS. The area covered by the blue box represents the area selected to average the healthy skin color, while the region covered by the red box represents the area chosen to average the color of the nevus. This image corresponds to a volunteer classified with skin phototype II.

Figure 4 shows the regions measured with DRS for the nevus and healthy skin of a volunteer classified with skin phototype II, where specific areas to obtain the average color value are highlighted in red for the nevus and in blue for healthy skin.

### 2.6. Spectral Data Preprocessing

The spectral data obtained by the previously described setup were normalized using the “zscore” function of MATLAB^®^ R2022 to ensure an adequate comparison between the experimental spectra of spots and nevi, and also between nevi and spots measured at different moments, by eliminating systematic variations or unwanted factors that may affect data comparability [38,39,40]. The normalized spectra were adjusted to have a zero mean and standard deviation equal to one, reducing the influence of outliers with respect to the differences in magnitude and experimental conditions variability. These normalized data were used for the calculation of the mean squared error (MSE), previously averaging the five normalized spectra taken for each selected zone either for the whole spectrum or for 3 specific spectral regions (region B, from 400 to 470 nm; region G, from 471 to 570 nm; and region R, from 571 to 750 nm), and also to perform principal component analysis (PCA) for comparing the similarity of the spectra of banana spots and skin nevi, as described in Section 3. All these algorithms were implemented in MATLAB^®^ R2022.

### 2.7. Simulation of Sampling Volume

**Figure 5:**
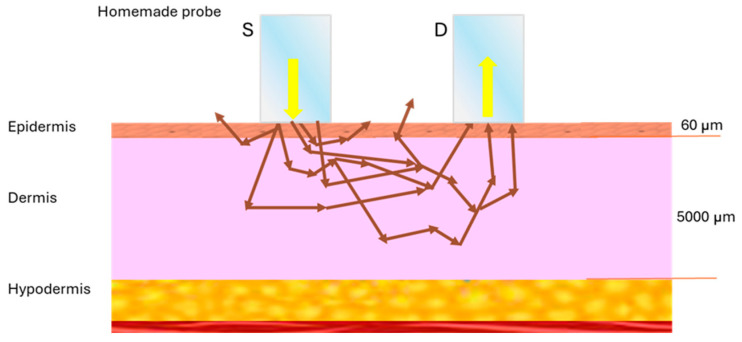
Main layers of the skin. The simulation of the sampling volume for both probes considers two main skin layers. The first layer corresponds to the epidermis, with a thickness of 60 microns, and the second layer represents the dermis, with a thickness of 5000 microns. ‘S’ and ‘D’ stand for “source fiber” and “detector fiber”, respectively. Yellow arrows show the direction of energy emitted from the source fiber into the sample and collected by the detector fiber, while brown arrows illustrate an example of photons’ paths within the tissue.

The simulation of the spatial distribution of detector sensitivity (sampling volume, SV) is described in the report by Meglinski and Matcher [34]. In essence, the objective is to calculate the average path, < l(r_m_)>, of all photons traversing a voxel of rm coordinates within the scattering tissue as they are transported from the source fiber (SF) to the detec-tor fiber (DF). We performed simulations based on a model of the SV of the two fiber-optic probe geometries used experimentally in this research using the “Cloud Monte Carlo” computing platform [41] described in the paper by Doronin and Meglisnki [42], by selecting the “Sampling Volume” application. Each probe consists of two identical optical fibers (980 µm and 200 µm diameter) arranged parallel to each other and separated by distances of 2500 µm and 240 µm between the centers of their cores, respectively. The simulation is performed in three configuration steps: (1) the scattering medium, where the optical parameters and the thickness of the skin layers are introduced. We considered only two layers, resulting in a relatively simple but useful optical model of the human skin, with 60 µm thickness for the first layer (epidermis) and 5000 µm thickness for the second layer (dermis); see Figure 5. (2) The source–detector geometry, where we specify the diameters of the SF and DF cores, the separation between the centers of their cores, and the acceptance angle of the optical fiber. (3) The extensions and dimensions of the output network, where the minimum and maximum values along the X and Y axes of the coordinate system of the two-dimensional quantity, < l(r_m_) > = Q(x,z), is recorded. Our objective was to examine the behavior of the sampling volume of the two probes employed in the spectral range of 400–750 nm for a discrete number of wavelengths: 400, 420, 500, 542, 578, 600, 650, 700, and 750 nm. The physiological parameters of the two skin layers were estimated to be representative, and the values utilized in the existing literature were adopted [43]. The website [44] provides information on creating an optical properties library in MATLAB^®^, where we specified the optical properties of the skin model above for the nine values of wavelength listed above. This simulation evaluates the SV contributing to the diffuse reflectance spectra measured experimentally, with each optical probe used for the healthy areas of the skin of volunteers. The physiological and scattering parameters used for each skin layer appear in Table 1.

The absorption coefficient, µ_a_ (λ), is defined by the various parameters presented in Table 1, where B is the blood volume fraction, S the oxygen saturation of hemoglobin, W is the water volume fraction, F is the fat volume fraction, and M is the melanosome volume fraction. On the other hand, the scattering coefficient, µ_s_ (λ), is calculated by the following parameters: µ_s.500nm_^’^ is the reduced scattering coefficient at 500 nm, f_Rayleigh_ is the Rayleigh scattering fraction at 500 nm, bMie is the Mie scattering power, and g is the anisotropy factor.

## 3. Results

The potential use of brown banana spots as biological phantoms of pigmented skin lesions requires the analysis and comparison of the main characteristics of both lesion types. Optical diagnostic techniques could take advantage of this novel approach of phantoms for skin pigmented lesions employing either colorimetric characteristics, purely spectral fingerprints, or even more advanced analytics by using dimensionality reduction approaches, such as principal component analysis. All these different characteristics are obtained and analyzed in this section in a comparative fashion for both lesions.

### 3.1. Analysis of the General Characteristics of the Spectral Response of Nevi and Spots

First, the general spectral characteristics of biological human skin lesions and brown banana spots are obtained and analyzed.

#### 3.1.1. Spectra Obtained in Spots on the Skin of the Banana Fruit

**Figure 6:**
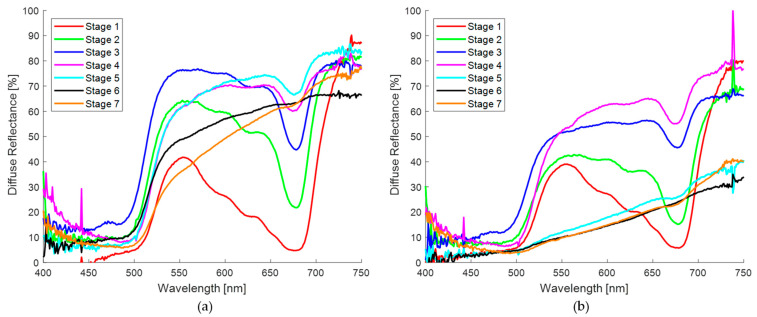
Diffuse reflectance spectra of banana fruit skin in two measurement regions with the fiber-optic probe with the largest distance between the centers of the emitting and collecting fibers (homemade probe): (**a**) Spectral curves of seven ripening stages of an area without a spot of banana fruit skin; (**b**) spectral response of an area with a spot at different ripening stages.

The diffuse reflectance spectrum responses expressed in percentage of the two regions of interest in the banana skin, taken with the homemade probe, are shown in Figure 6. These regions correspond to the seven ripening stages of the banana according to the visual aspect of the samples used in this experiment. In Figure 6a,b, low reflectance can be observed in two spectral regions, 400–500 nm and 600–700 nm, being particularly remarkable in the banana skin, with a reflectance valley at 680 nm. In addition, a high reflectance of green light (550 nm) is observed, as expected based on the apparent color of the samples at early stages, and at 720 nm as a consequence of the low absorption of the plant tissue [45]. On the other hand, the diffuse reflectance in the spots (Figure 6b) is lower overall compared to the region without them (Figure 6a).

**Figure 7:**
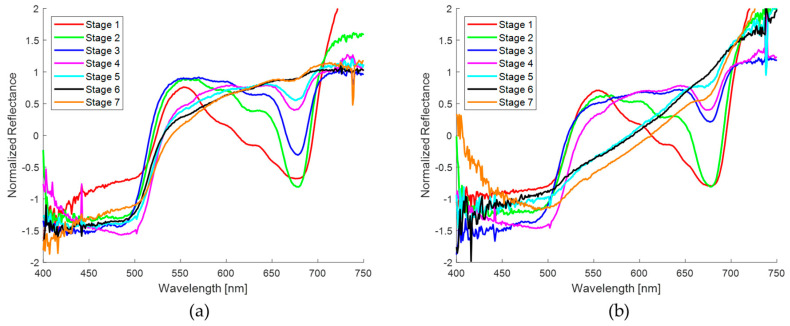
Normalized averaged spectral curves obtained from two regions studied on the skin of the banana fruit for samples in seven stages of maturation: (**a**) Spectra of the skin without the presence of the spots or lesions in a region near to the selected spot, and (**b**) spectra of the brown spots selected in the same sample.

Figure 7 presents the normalized diffuse reflectance spectra of the two regions of interest in the banana skin described above. As can be seen, the normalization allows for a more accurate comparison between the experimental spectra of both regions of the skin. In Figure 7a,b, the same behaviors previously described are observed, showing a lower diffuse reflectance in the measurements performed on the spots (Figure 7b), confirming the spectral differences associated with the surface characteristics of the banana skin.

**Figure 8:**
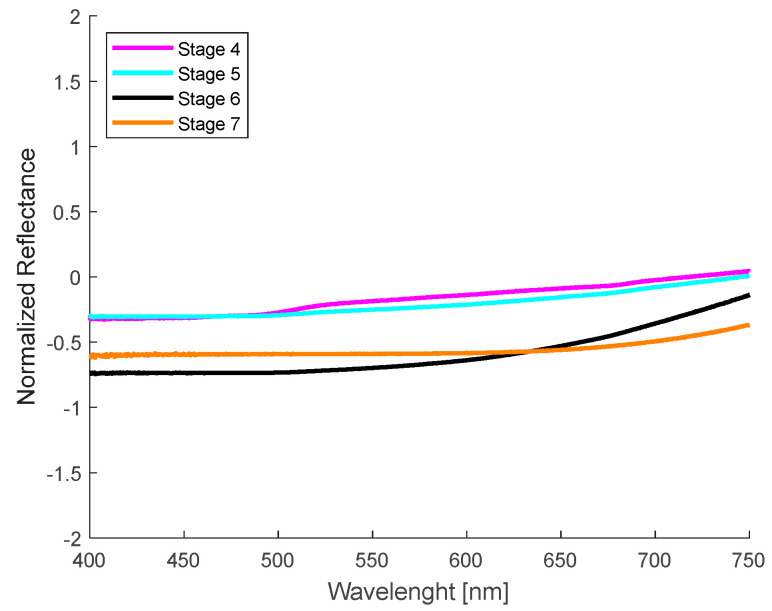
Normalized spectral curves recorded with the commercial probe on banana fruit skin spots at four ripening stages (4 to 7).

Figure 8 shows a representative sample of the normalized spectral curves obtained from the spots on the skin of banana fruit recorded with the commercial probe. Due to the analysis of the results with the homemade probe, it was decided to limit the study to the ripening stages between 4 and 7, this was due to the fact that the dimensions of the spots in these stages allow for the exclusive analysis of the pigmented zone with the commercial probe. It can be observed that a very low diffuse and almost constant reflectance is obtained in the whole spectral region analyzed. This result is in clear contrast to the measurements with the homemade probe, which showed the presence of reflectance valleys at 680 nm. A comparison of the spectra obtained with both probes revealed a notable discrepancy despite the samples under evaluation exhibiting a comparable maturation stage.

The spectra of the regions of the banana skin without spots obtained with the commercial probe were excluded from the analysis, as this study focuses on analyzing the spectral similarity of the pigmented zone only.

#### 3.1.2. Spectra Obtained in Nevi and Healthy Skin

**Figure 9:**
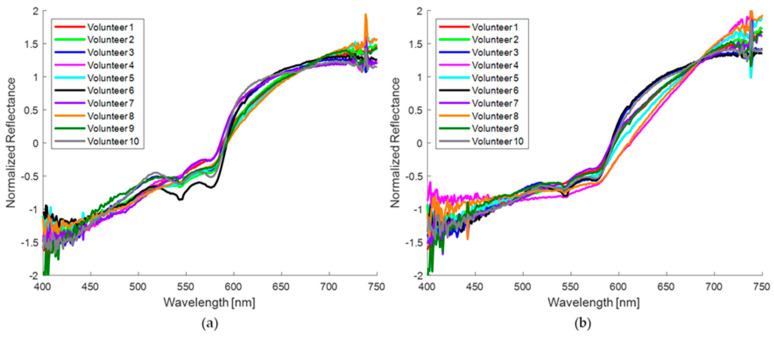
Spectral normalized curves obtained in two skin regions of 10 volunteers: (**a**) Spectra corresponding to a skin region close to the nevus; (**b**) spectra corresponding to the selected nevi (one per volunteer).

Normalized diffuse reflectance spectral curves of healthy skin and nevi of 10 volunteers taken with the homemade probe are shown in Figure 9a,b, respectively. Figure 9a shows slightly higher reflectance compared to the corresponding pigmented region (Figure 9b). All spectra in healthy skin show two reflectance valleys between 500 and 600 nm that are typical of oxyhemoglobin, commonly reported as a “W” shape. Hemoglobin is one of the key chromophores for the optical characterization of the skin, as well as the presence of melanin, which is characterized by reduced reflectance in the UV–visible range. In addition, water constitutes 60–70% of the skin; however, its influence in the visible range is minor, with a subtle impact near 700 nm. It is essential to consider the influence of these chromophores in the absorption and scattering of light.

**Figure 10:**
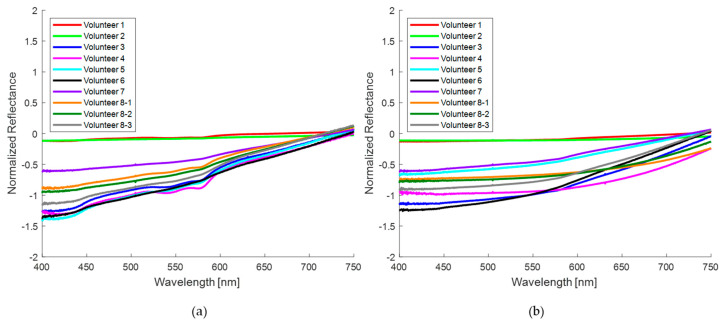
Normalized spectral curves were obtained from two regions of interest from eight volunteers: (**a**) Spectra taken in a region of healthy skin in close proximity to the nevus, and (**b**) spectra corresponding to the nevi measured in each volunteer. In the case of volunteer 8, three measurements were taken, designated as “8-1”, “8-2”, and “8-3”, corresponding to the order in which they were taken.

Figure 10 shows the normalized spectral curves for healthy skin (Figure 10a) and for nevi (Figure 10b) of the eight volunteers measured with the commercial probe. It is evident that the distinctive “W” shape is almost absent in most of these measurements for both regions under consideration. Furthermore, the measurements obtained with this probe demonstrate a reduced reflection across the entire spectral range in comparison to the homemade probe. This discrepancy can be attributed to the differing sampling volumes of the two probes. A smaller probe size limits the amount of reflected light, thus reducing the sampling volume. This aspect is addressed in more detail in the discussion of this work.

In general, for both probes the reflectance in nevi is lower than the reflectance in healthy skin due to the presence of melanin that absorbs the incident light [46]. However, this is not the only cause since the amount of reflected light also depends on the skin phototype of each subject volunteer, and also on the area of the body where the measurements are taken. The measurements with the homemade probe were performed at the wrist (volunteer 5), back of the hand (volunteers 3 and 9), palm (volunteer 10), arm (volunteers 4 and 7), forearm (volunteers 1, 2, and 6), and fingers of the hand (volunteer 8); while for the commercial probe, the measurements were carried out at the wrist (volunteer 3), back of the hand (volunteers 5 and 7), palm (volunteers 1 and 4), forearm (volunteers 2, 6, and 8-2 and 8-3), and neck (volunteer 8-1).

### 3.2. Morphological and Colorimetrical Characteristics of the Spots and Nevi

With the general spectral fingerprints of both lesions having been presented, in this Section 3.2 morphological surface characteristics and colorimetric fingerprints are obtained and analyzed.

#### 3.2.1. Areas of Nevi and Spots

**Figure 11:**
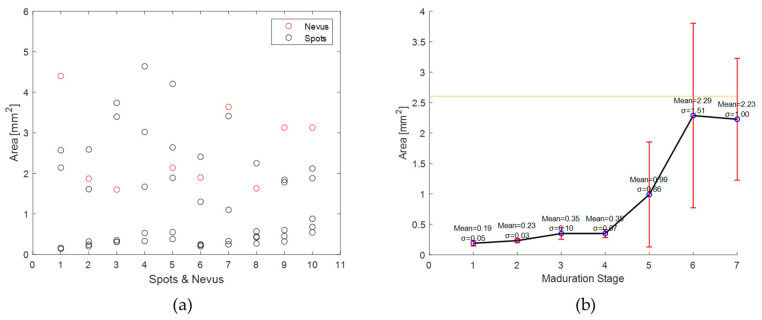
(**a**) Areas of the volunteers’ nevi and brown spots on the skin of the banana fruit. The red circles represent the nevi of the 10 volunteers (volunteer 4 was excluded due to the large area of their nevus, 42.43 mm^2^). Black circles represent the selected spots in the different banana fruit samples. (**b**) Graph showing the average area of the brown spots according to the ripening or maturation stage of the sample, where the yellow line shows the value of the average area of the nevi (in this calculation the nevus of volunteer 4 was excluded, as indicated before).

The areas (A) in mm^2^ of the nevi and brown spots measured with the homemade probe are shown in Figure 11a, where the red circles represent the nevi and the black circles the brown spots. The nevus of volunteer 4 was not included because its area is very large compared to the rest (42.43 mm^2^). As can be seen in Figure 11b, the area of the spots grows as a function of the stage of the samples at the moment of carrying out the DRS measurements. The areas during maturation stages 1 to 4 are quite similar (0.23–0.37 mm^2^), and quite far from the average area of the nevi (2.61 mm^2^). In the case of the spots evaluated in the samples at maturation stages 5, 6, and 7, their areas (0.99–2.23 mm^2^) are similar to the average area of the nevi, showing a higher standard deviation. This is due to the fact that, in the early stages, the growth of the spots is slow due to the high presence of chlorophyll. On the contrary, in the advanced stages, yellow or brown spots are formed, which vary in size as the ripening process progresses, tending to join together, generating greater variability.

#### 3.2.2. Analysis of Color Intensity

**Figure 12:**
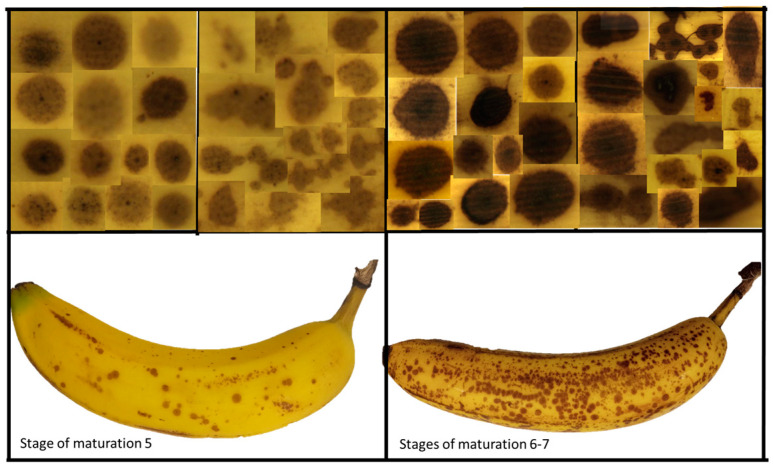
Images of spots on the skin of banana fruit taken from samples of different ripening stages.

Visual information is of utmost importance in the analysis and diagnosis of pigmented skin lesions. Non-invasive techniques, such as confocal microscopy and polarization-sensitive optical coherence tomography (PS-OCT), permit the acquisition of images with high resolution and contrast, facilitating the accurate assessment of the characteristics of the lesions and enabling the monitoring of changes in them [47,48,49]. Multispectral and microscopic images provide valuable data on distribution, composition, and morphological features, which are useful for differential diagnosis [50,51]. However, it is known that scientific and medical research often encounters obstacles in obtaining human or animal samples for studies due to various constraints. Simulators (phantoms) are presented as an alternative, but they can be expensive and slow to acquire. For the above reasons, the present study suggests the use of spots on banana samples as a possible auxiliary or phantom in biomedical optics for the validation and training of non-invasive optical techniques based on the acquisition and analysis of digital images. This proposal reinforces the feasibility of the use of banana skin spots. As an example, Figure 12 shows some images of banana skin spots taken from samples at different stages of ripening, where the variability in their dimensions, shapes (regular and irregular), and level of pigmentation can be appreciated. These images could serve as a basis for training images to enhance the precision of edge segmentation algorithms. It is well known that lesion borders are often diffuse or irregular, which complicates border recognition, being one of the most challenging steps in the segmentation process, with the accuracy of subsequent stages directly influenced by the outcome of this initial stage [52,53,54]. It is therefore beneficial to have extensive digital image databases to improve the accuracy of algorithms and clinical results, which could reinforce the idea of employing images of banana skin spots to train algorithms to detect features such as borders, symmetry, and tonalities, characteristics that are crucial in the delineation of pigmented lesions.

**Figure 13:**
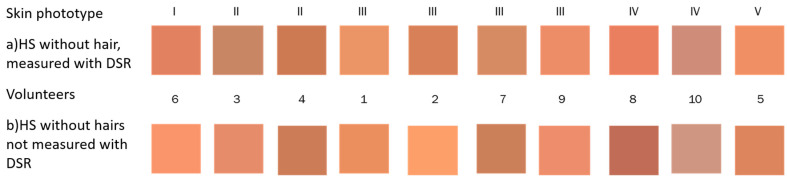
Results of the analysis of the average value of the color intensity of each volunteer ordered by skin phototype according to Fitzpatrick’s classification (see Figure 3), after evaluating two skin regions, from the results of the Silonie Sachveda survey. (**a**) The first row corresponds to a visually selected area of healthy skin (HS) without the presence of lesions or hair, measured with DRS; (**b**) region of healthy skin (HS) under similar circumstances in which no DRS measurements were performed (each volunteer is referred by the number 1–10).

Some optical techniques rely on color information to give a diagnosis. As a consequence, a script was implemented to analyze RGB components of human skin nevi and banana spots. First, the repeatability of the calculation at different locations was analyzed. The average value of color intensity was calculated in two regions of healthy skin of the same volunteer; see Figure 13. The values obtained for each color channel (channel R—red, channel G—green, channel B—blue, all belonging to the RGB system) are presented in Table 2, where the difference between both locations is quantified by means of the relative percentage error (Er). Color images for each volunteer on healthy skin as a function of phototype can be seen in Figure 13. In this figure, it can be observed that there is a great similarity of color tones between the two regions. These results were quantitatively corroborated by calculating the relative percentage error Er(%) for each RGB channel. This analysis results in most of the errors being less than 10%, as shown in Table 2. For example, volunteers 9 and 10 show a very small relative percentage error in the three channels (0.45% < Er(%) < 3% and 2% < Er(%) < 7%, respectively), with their tones being visually equal (see Figure 13). On the other hand, slight differences appear in color tone in some of the volunteers where the relative percentage error is greater, reaching values higher than 18% (volunteers 2 and 8). This fact is also reflected in Figure 13, where the colors are visually different.

**Figure 14:**
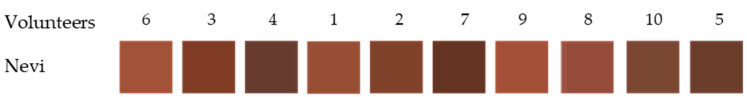
Shades obtained for the volunteers’ nevi as a result of the calculation of the average value of the color intensity in the RGB system.

Due to the good performance of the developed algorithm, the average value of the color intensity for each channel of the RGB system of the 10 nevi and the 48 spots was calculated. Figure 14 shows the color shades obtained for the ten nevi, ordered according to the skin phototype of each volunteer. To evaluate the differences between the color shades of the spots with respect to the nevus, the relative percentage error Er(%)_N_ was calculated for each nevus–spot pair in each RGB channel (1440 combinations), taking the nevus as the true value. Then, the average color value between the three numerical Er(%)_N_ values of the RGB channels of each nevus–spot pair (ARE) was calculated, giving a total of 480 values. From these, only the combinations with the lowest ARE value were selected and the spots that most resembled the nevi of the volunteers in this study could be identified. When comparing the shades obtained with Figure 13, that represents healthy skin, a darker appearance is appreciated, as expected. Table 3 shows all the information regarding the brown spot with the lowest ARE for each nevus. Data on the ripening stage of the brown spots are also included.

**Figure 15:**
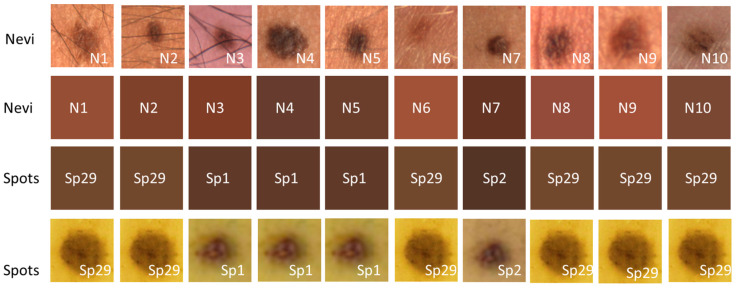
Comparison of the average color of nevi, (N1–N10) and spots (Sp1, Sp2, and Sp29) in the banana fruit skin, where the minimum value of ARE between the three relative percentage errors was obtained for each nevus–spot combination. The spots with the greatest similarity were captured in samples with the higher ripening stages: 4, 5, and 6. Furthermore, the actual images of the nevus and the spot are positioned in the top and bottom rows, respectively.

The resulting shades for the spots were generally darker than the color shades obtained for the nevi of the volunteers in this study. For most of the combinations performed, values between 40% and 50% were obtained from the ARE calculation, especially with spots evaluated at a low stage of maturation. Only in 7.29% (35 combinations) of the comparisons made was it less than 20%, with these belonging mostly to comparisons made with the nevi of volunteers 2, 3, 4, 5, and 7. According to the results shown in Table 3, the spots with the highest similarity to nevi were three (Sp1, Sp2, and Sp29) with a high maturation stage (S4–S6). Especially in the case of the Sp29 spot, we can appreciate that it is comparable with most of the nevi (six comparisons), particularly with the nevi of volunteers classified with skin phototypes III and IV. It should be noted that this spot was evaluated in a sample with a high maturation stage (S6) and with an area of 2.57 mm^2^, which is similar to the average area of the nevi: 2.61 mm^2^. On the other hand, it is highlighted in that in Table 3 the ARE values are in a range between 4.51% and 17.53%, which indicates a good similarity between these spots and the analyzed nevi. The color shades of these ten combinations can be seen in Figure 15, where differences can be seen precisely in those with the highest ARE, between 13% and 17% (N1, N6, N8, and N9). The image shows four rows where the aforementioned combinations are arranged. For a better visualization, a cutout of the photograph of each area, both nevi and spots, respectively, has been presented. Therefore, we could establish an acceptable ARE value of less than 10% instead of choosing the minimum value, which allowed us to consider other combinations with darker spots and nevi that could also provide useful information. Specifically, these new combinations were N4 with Sp4 and N7 with Sp3, with ARE values of 10.11% and 8.30%, respectively. It should be noted that while the spots may show similarities in terms of overall ARE value, they do not necessarily have the same minimum error in each channel separately.

### 3.3. Spectral Comparison Between Nevi and Spots

#### 3.3.1. Mean Squared Error

The similarity between the spectral responses of nevi and spots to previously normalized spectra from 400 nm to 750 nm was determined by calculating the mean squared error (MSE). The results of the MSE values obtained were low, less than 0.2 in 55% of the 480 comparisons. For the values obtained, a threshold of 0.2 for the MSE was considered as the maximum acceptable difference between nevi and spots. Table 4 (left side) shows only the minimum MSE values for each nevus, where it is observed that all are of the same order of magnitude and in an interval between 0.02 and 0.07. In addition, the best results were obtained for selected spots in samples with maturation stages 5 or 6, with Sp23 being spectrally comparable to seven of the nevi in this study.

The MSE calculation was also performed in three specific regions of the diffuse reflectance spectrum, classified as R, G, and B. Region B includes a wavelength range from 400 nm to 500 nm, region G from 501 nm to 570 nm, and region R from 571 nm to 750 nm. The results of the obtained MSE values were lower than the selected threshold 0.2 in 40% of the 480 comparisons in the R spectral region, 22% in the G region, and 0.6% in the B region. Table 4 (right side) shows only the minimum MSE values for each of these R-G-B spectral regions. It is observed that the R region is the one with the best spectral correspondence between the nevi with the related spots, where all the MSE values are below the established threshold (0.2) and mostly even between 0.01 and 0.06. It can also be seen that spectral comparisons with the Sp29 spot, whose stage of maturation is 6, are predominant. However, for region G the correlation is good in five of the nevi where the MSE is less than 0.2. As for region B, the discrepancy is high, all MSE values except one are above the established threshold of 0.2. Note that the minimum values obtained for the MSE for each region do not necessarily coincide with the same spot.

**Figure 16:**
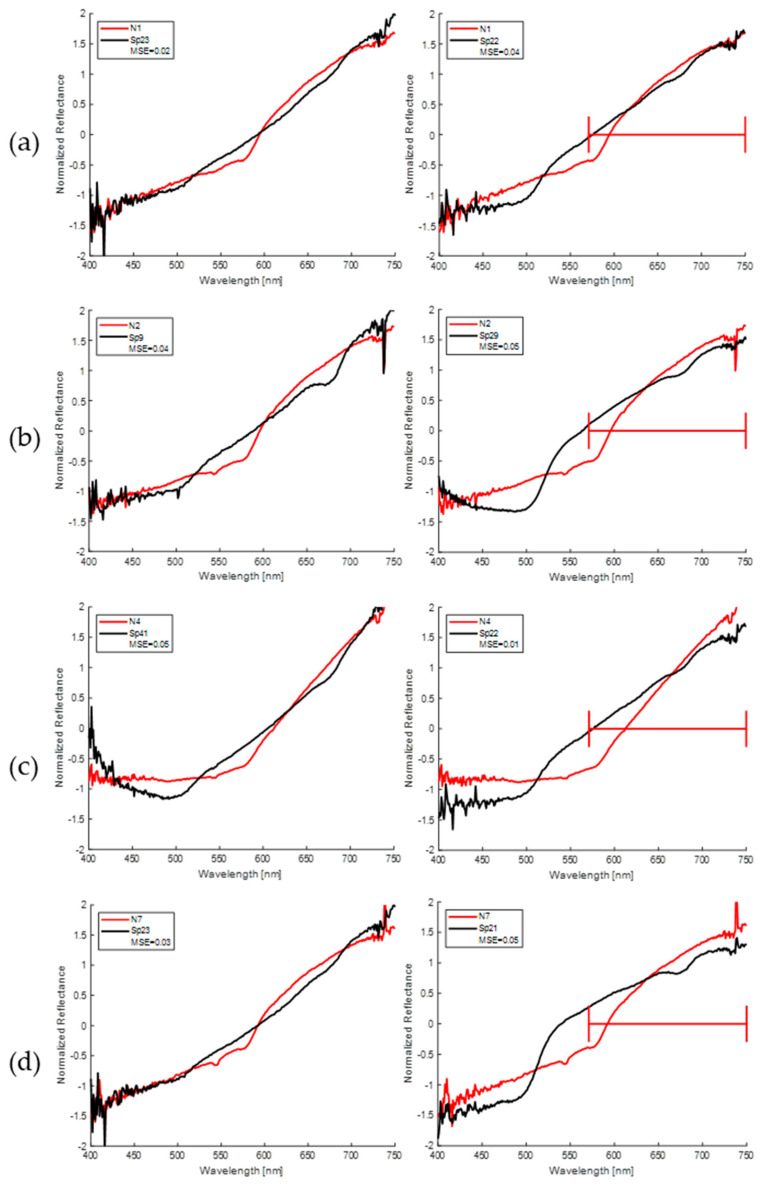
Spectral comparison between nevi and spots considering the minimum MSE values with the normalized spectra from 400 nm to 750 nm. The first column shows the general spectral comparison, while the second column presents the comparison of the nevus with the spots with the minimum MSE value for the spectral region classified as R (from 571 to 750 nm; range marked by a horizontal red bar in the figures): (**a**) Spectra of the nevus of volunteer 1 and the Sp23 spot, with an MSE of 0.02, and the Sp22 spot; (**b**) spectra of the nevus of volunteer 2 and the Sp9 spot, with an MSE of 0.04, and the Sp29 spot; (**c**) spectra of the nevus of volunteer 4 and the Sp41 spot, with an MSE of 0.04, and the Sp22 spot; (**d**) spectra of the nevus of volunteer 7 and the Sp23 spot, with an MSE of 0.03, and the Sp21 spot.

In order to have a qualitative view of the spectral similarity between nevi and spots with the minimum MSE, a representation of the full spectral region (first column) is shown in Figure 16, as well as for the R spectral region (second column) of the four combinations with the lowest MSE for the homemade probe. As can be appreciated, the visual similarity is quite significant for the whole spectrum, although there are some ranges with slight differences.

The same analysis technique was also applied to the spectral data collected with the commercial probe. In this analysis, minimum mean squared error (MSE) values were calculated from the spectral data for the new set of nevi and spots. Table 5 shows the minimum MSE values obtained for each of the nevi. It can be seen that the calculated MSE values were all much lower than the preset threshold (0.2), even an order of magnitude lower than those obtained with the homemade probe. This result is favorable because it indicates a greater similarity between the spectra evaluated for nevi and spots when using the commercial probe. According to Table 5, the stages of the spots with the lowest MSE were limited to stages 5 and 6, corresponding to high levels of maturation. In addition, the spot with the highest similarity to nevi was Sp18 (four combinations). It should be noted that this spot belongs to those classified as dim spots, that is, those with a light brown color, also we can notice a greater variety of spots with which the minimum MSE was obtained for this probe, that is seven different spots, in contrast to the four spots with which the minimum MSE was obtained for the homemade probe.

**Figure 17:**
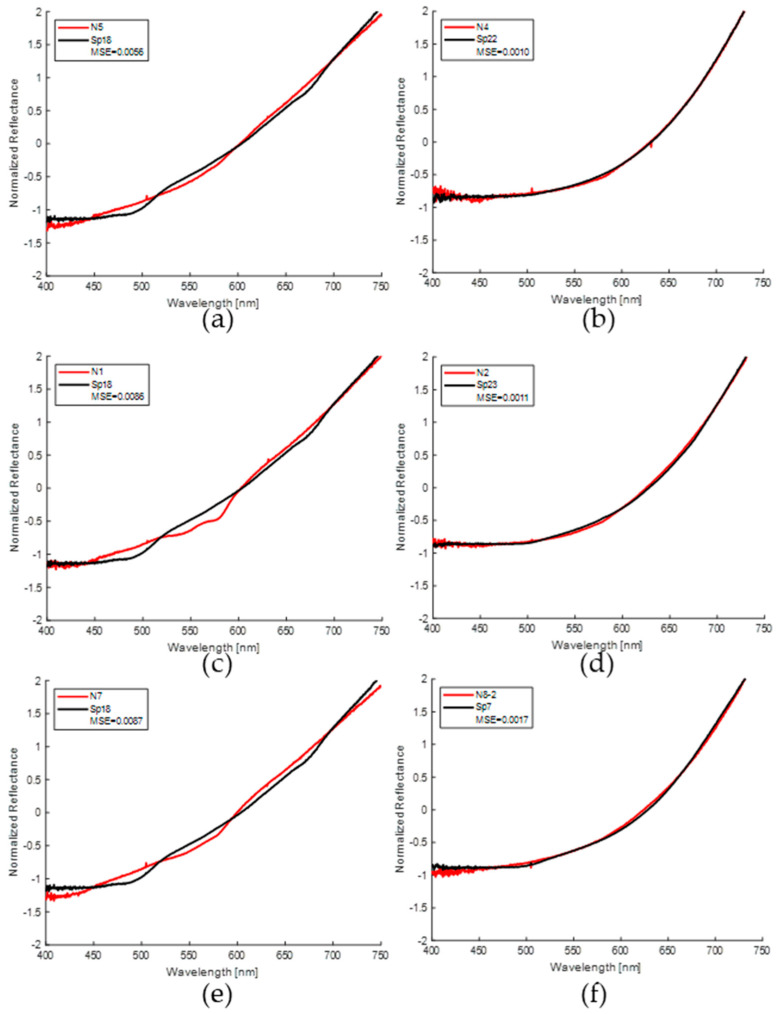
Spectral comparison between nevi and spots measured with the commercial probe, considering the minimum MSE values of the normalized spectra from 400 nm to 750 nm. The first column (**a**,**c**,**e**) presents the three combinations with the maximum MSE values obtained among the minima, while column two (**b**,**d**,**f**) shows the three combinations with the lowest MSE, highlighting mainly (**a**) nevus of volunteer 7 compared to Sp18 spot, with an MSE of 0.0087, being the maximum MSE value among the minima obtained; and (**b**) nevus of volunteer 4 compared to Sp22 spot, with an MSE of 0.0010, being the lowest value among those obtained with the commercial probe.

The comparison of the normalized spectral curves between nevi and spots with the minimum MSE value shows a better agreement throughout the spectral range for the measurements made with the commercial probe. These results are presented in Figure 17. However, although in most nevus–spot combinations the similarity is appreciable, in some nevus spectra the “W” shape is still prominent (volunteer example 1), which generates a slight difference due to the presence of hemoglobin in this spectral region. The right column of this figure shows the spectra of the nevi vs. spots with the three lowest MSE values, while the left column shows the spectral curves with the three highest values.

#### 3.3.2. Principal Component Analysis and Mahalanobis Distance

**Figure 18:**
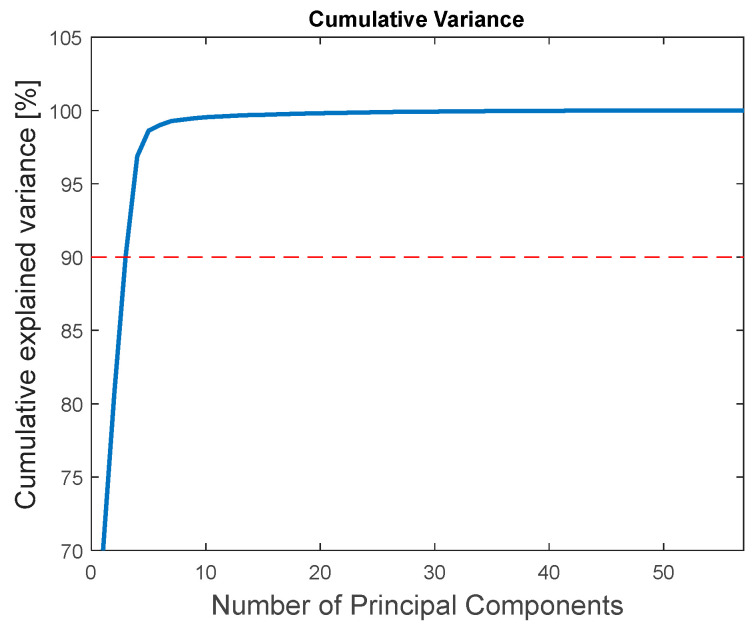
Cumulative variance explained by different numbers of principal components of nevi and spot spectra. The dashed line indicates the 90% variance threshold, which is obtained from the third component on.

The calculation of the principal components of the normalized spectral data of nevi and spots was performed using MATLAB’s Statistics and Machine Learning Toolbox. First, the variability of the PCA was analyzed in order to estimate a reasonable number of PCA components to be considered. The variance explained by the model is 98.63% for 5 components and 99.53% for 10 components, as can be seen in Figure 18. The analysis indicates that the dataset could be reasonably explained with as few as five components. Consequently, the analysis is made with 5 and 10 components.

In order to make a comparison between the principal components of nevi and spots, first the Euclidean distance of the PCA components of each pair was calculated. However, the results were distorted by the differences in absolute magnitude for some bigger PCA components, compared with others with more relative differences but smaller absolute magnitude. In order to avoid this problem, the analysis was made by calculating the Mahalanobis distance. The Mahalanobis distance considers the centroid of the multidimensional PCA component space, and the spread of each PCA component. The results of the calculation appear in Table 6.

The calculation of the Mahalanobis distance with data obtained from the first five PCA components shows short distances as a result, as can be appreciated in Table 6. This fact suggests a good similarity between the samples studied. The minimum values of the distances obtained for each of the nevi are coincident with spots Sp23 and Sp24. These spots correspond to ripening stages 5 and 6, respectively. The analysis of 10 PCA components shows that the Mahalanobis distances increase; see Table 6. In this last case all the nevi resemble spot Sp23, at maturation stage 5.

**Figure 19:**
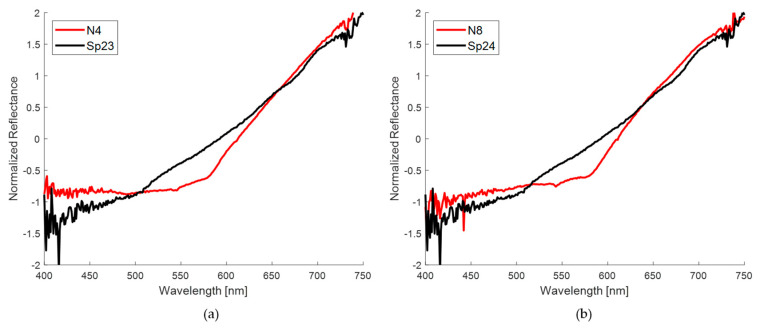
Spectral comparisons between nevi and spots according to the minimum values of the Mahalanobis distance, calculated from the first five principal components. (**a**) Comparison between the normalized spectra of the nevus of volunteer 4 and Sp23 spot; (**b**) comparison between the normalized spectra of volunteer 8’s nevus and Sp24 spot.

Although the visual spectral similarity is not so relevant for this PCA analysis, Figure 19 shows examples of the whole spectral comparisons of nevus and spot pairs, according to the calculation of the minimum Mahalanobis distance. As expected, the similarity is not so close as with the previous direct spectral analysis.

### 3.4. Fiber Probe Sampling Volume Simulation

**Figure 20:**
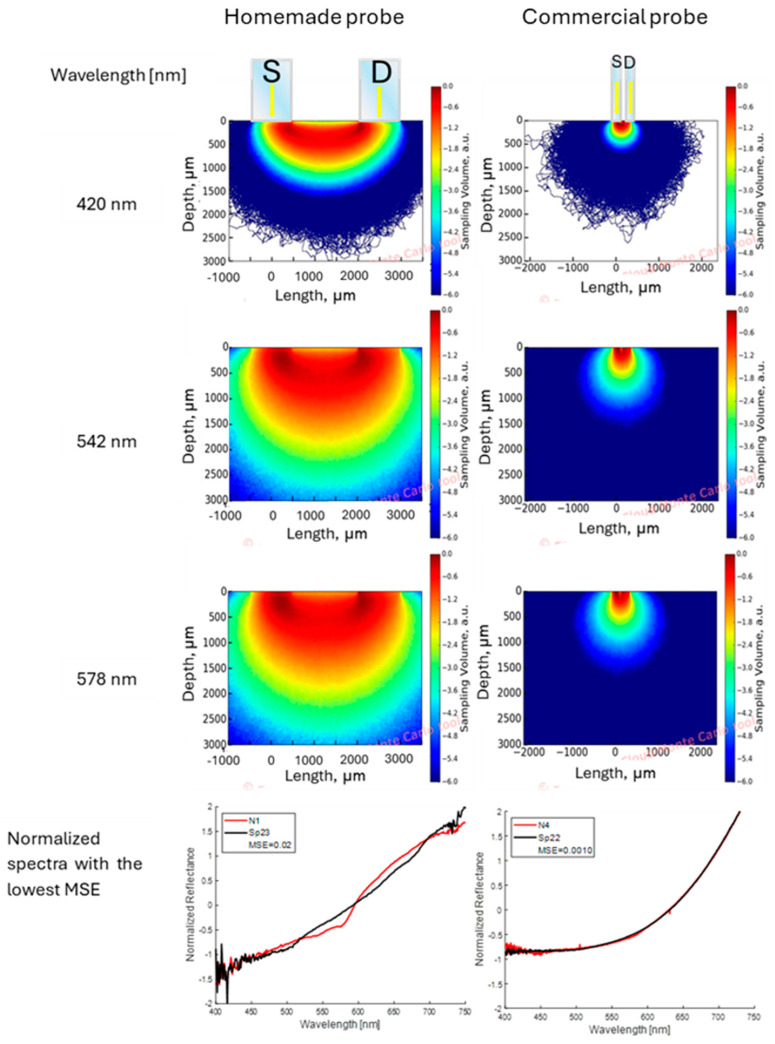
A comparative study of the SV simulation of both probes for light with wavelengths representative of the spectral range of measurement and its impact on the similarity of diffuse reflectance spectra of pigmented lesions of human skin and brown spots on bananas.

Figure 20 shows the results of the sampling volume (SV) simulation for the two optical fibers used considering the skin model described above. The results are shown for three characteristic wavelengths (λ = 420, 542, and 578 nm) in the measurement spectral range 400–750 nm. The left column of the figure shows the SV simulation result for the homemade probe (source–detector distance = 2500 μm), while the right column presents the results for the commercial probe (source–detector distance of 240 μm). It is crucial to highlight that, in all simulations conducted, both probes exhibited a high degree of spatial symmetry in the distribution of the SV, Q(x,z), which can be attributed to the highly symmetric configuration of the probes and the scattering medium. This includes equal inclination angles of the fibers of each probe with respect to the surface normal, the utilization of the same type of material, and the identical diameters of the fiber cores. A scattering medium comprising two homogeneous layers was used. A noteworthy decline in SV is evident for the commercial probe in comparison to the homemade probe across all wavelengths. This is attributed to the limited source–detector distance (240 μm) and the reduced core size of the two optical fibers (200 μm) utilized in the commercial probe. Additionally, it is observed that the SV for both probes is highly similar at wavelengths λ = 542 nm and 578 nm, which correspond to the maxima of the molar extinction coefficient of oxygenated hemoglobin [55]. Furthermore, the lowest SV is observed at λ = 420 nm, likely due to the high absorption coefficient relative to the reduced scattering coefficient in the dermis at that wavelength. Finally, a very remarkable issue is that the bottom of Figure 20 illustrates a superior alignment between the diffuse reflectance spectrum of pigmented skin lesions and brown spots on bananas when the commercial probe is utilized, in comparison to the homemade probe.

**Figure 21:**
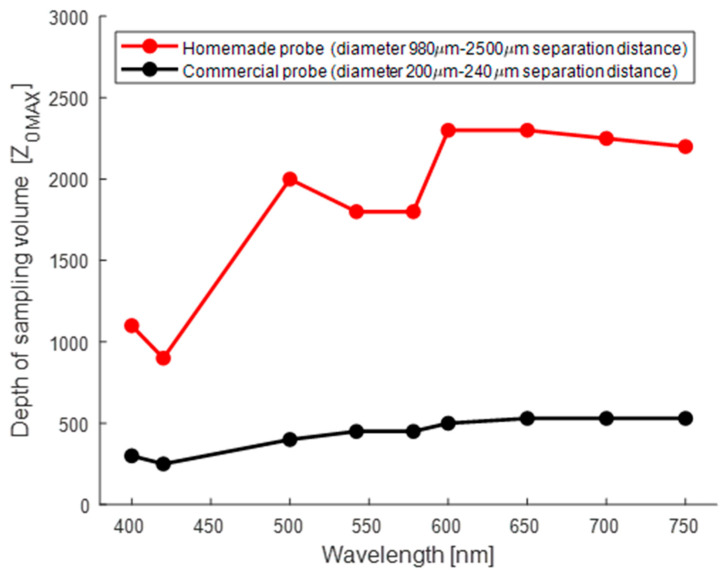
Spectral dependence of the maximum depth of the sampling volume, Z^0^_Max_, for the two fiber-optic probes employed in this study using nine discrete wavelengths in the spectral range of interest.

Figure 21 illustrates the spectral behavior of the maximum depth, Z^0^_Max_, of the SV for both probes. It is observed that for the commercial probe the smallest sampling volume coincides with the wavelength λ = 420 nm, resulting in a maximum depth of Z^0^_Max_ = 250 μm. From that wavelength, there is a slight increase in the maximum depth, reaching a constant value zone in the spectral region of 650–750 nm, with a maximum depth of Z^0^_Max_ = 530 μm. In contrast, the homemade probe does not exhibit a discernible trend. However, similar to the commercial probe, the smallest sampling volume is observed at the wavelength λ = 420 nm, with a maximum depth of approximately 900 μm. From the values for Z^0^_Max_ of the sampling volume for both probes, it can be observed that the coefficient C, defined as Z^0^_Max___Commercial/_Z^0^_Max___Homemade_, satisfies the condition Z^0^_Max___Commercial_ ≤ 0.28* Z^0^_Max___Homemade_ for all wavelengths at which the simulation was performed. The lowest value for C was 0.2, obtained for λ = 500 nm, which coincides with one of the seven isobestic points of oxy- and deoxyhemoglobin (422, 452, 500, 529, 545, 570, and 584 nm) within the relevant range of 400–750 nm [56]. It is important to note that even for the commercial probe, the SV extends beyond the thickness of the epidermis (60 μm). Consequently, the signal contributing to the diffuse reflectance spectrum contains information on the presence of both melanin in the epidermis and blood at depths between 300 and 530 μm in the 400–750 nm spectral region.

Figure 21 illustrates the presence of local minima at 542 and 578 nm for the maximum depth of the sampling volume, which coincides with the maximum absorption peaks in the visible spectrum of hemoglobin. However, a more detailed explanation of the possible reasons behind the behavior of the sampling volume in these areas is presented in Section 4.

## 4. Discussion

In this work, the potential use of banana skin brown spots as phantoms of pigmented human skin lesions was evaluated. The ideal properties of a phantom are highly dependent on its intended use and must meet certain characteristics such as accuracy, stability, and reproducibility. In addition, optical (absorption and scattering) and mechanical properties similar to those of the tissue to be replaced are required. Ease of manufacture, cost, and portability are also important considerations. These characteristics are compared and discussed in the evaluation of different optical phantoms [57,58,59] which are practically equivalent to having a ‘standard sample’. However, in this work we set out to investigate under what experimental conditions (including, among other variables, the dimensions, tonality, and maturation stages of the banana brown spots) a degree of similarity could be established between the spots and the pigmented skin lesions of the group of recruited volunteers. In this work, we are not claiming that our proposed phantom is a ‘standard sample’, but that at certain stages of the banana ripening maturation process, brown banana spots can be used with an acceptable similarity, in terms of dimensions, tonality, and diffuse reflectance spectrum, to pigmented skin lesions. During these time periods, although short compared to a standard phantom, it is valid to use our proposed pigmented lesion mimic to test the developed diagnostic algorithms, which are generally based on different optical techniques.

First, the general diffuse reflectance spectra of banana brown spots and human skin nevi were presented. Figure 6b shows the diffuse reflectance spectra measured with the homemade probe sorted by maturation stage. The normalized spectra are shown in Figure 7b and Figure 8 for measurements made with the homemade and commercial probes, respectively. Finally, Figure 9b and Figure 10b show the normalized spectra of the nevi measured with both probes. When comparing the areas of the banana without a spot and with a spot (Figure 7a,b), low reflectance is observed in two spectral regions of 400–500 nm and 600–700 nm, mainly due to the presence of chlorophylls that absorb light in these wavelength ranges [45]. These results are in agreement with those reported by Meng Li et al., Zude et al., Subedi et al., Rajkumar et al., Hashim et al., Wang et al., and Xie et al. [26,60,61,62,63,64,65], who emphasize the presence of an absorption band at 680 nm, especially when the banana skin is visibly green (ripening stages 1 and 2). These analyses support the idea that the presence of chlorophyll is a good indicator for the classification of the ripening stage. Previous research has focused primarily on determining the quality and shelf life of banana fruit using non-invasive optical techniques such as spectroscopy and multispectral and hyperspectral imaging. On the other hand, the diffuse reflectance in the spots (Figure 7b) is generally lower compared to a region without spots (Figure 7a). This behavior is very similar to that recorded at 20 °C at six ripening stages by Rajkumar et al. [62]; and to the spectra of bananas with four levels of browning (no browning, slight, moderate, and serious) reported by Wang et al. [64].

Because the selected spots are smaller than the probe area of the detector fiber used by the homemade probe, the reflectance valley at 680 nm is more pronounced for the first ripening stages (1–2), similar to the valley around 630 nm, due to the high concentration of chlorophylls (Figure 7b). As the green hue of the banana skin begins to disappear as a result of the rapid chlorophyll degradation process, starting at the fourth stage of ripening, the shape of the spectrum changes in the range from 500 to 680 nm. As the spot grows and darkens, the diffuse reflectance decreases and the curves resemble a straight line. This last behavior corresponds to the higher stages of maturation in this study: 5, 6, and 7. In the spectra obtained with the commercial probe, which has smaller-diameter optical fibers, probing was guaranteed only in the pigmented region of the spots, so that the characteristic valleys of chlorophyll are imperceptible [66], in addition to presenting a low reflectance, as can be seen in the spectral curves presented in Figure 8. These curves are similar to the spectra reported by Wang et al. [64] in samples with a high degree of browning.

When analyzing the diffuse reflectance spectrum of human skin, distinctive features related to the presence of oxygenated hemoglobin are apparent. This is manifested in the recognizable “W” shape in the spectrum, which shows reflectance minima around 542 nm and 575 nm, attributed to the absorption of oxygenated hemoglobin [67]. While the characteristic “W” shape is more pronounced in the measurements obtained with the homemade probe, as shown in Figure 9a, and even in some nevus spectra obtained with the same probe (Figure 9b), for the spectra collected with the commercial probe, this characteristic shape is almost imperceptible in most of the normalized spectra, as shown in Figure 10a.

When a qualitative comparison is made between a banana region without spots (Figure 7a) and the healthy skin of volunteers (Figure 9a), noticeable spectral differences are observed. However, with the attenuation of the “W” shape in the human nevi (Figure 9a,b), or the absence of the reflection valley at 680 nm for the banana skin (Figure 7a,b), the spectra begin to look similar. When contrasting the banana spots (Figure 7b) with human nevi (Figure 9b), it is evident that maturation stages below 5 still show a pronounced valley at 680 nm and an increase in reflectance from 500 nm (Figure 7b), which is not observed in human nevi (Figure 9b). However, this behavior is not observed in the measurements made with the commercial probe, since the measured reflectance is consistently low, resulting in a greater similarity between the spectra of nevi and spots. The variation in the shape of the diffuse reflectance curve obtained for both probes is attributed to multiple factors, such as the presence of melanin, the skin phototype, and the regions examined, as well as the geometry of the probe and its characteristics (fiber diameter and distance between them). These purely qualitative comparisons show that the ripening stage of the banana fruit is a significant parameter for its evaluation as a potential phantom, at least for diagnostic techniques based on direct spectral shape.

The quantitative evaluation of the potential of the proposed phantom requires the analysis of morphological, colorimetric, and spectral characteristics. The area of banana and nevus spots was estimated using an algorithm and compared in Figure 11. Figure 11a shows that there is greater variability in the area of banana spots (dark circles), which can a similar size to nevi or smaller. Figure 11b shows that this is a function of ripening stage, as expected, as the area of the spots increases with increasing ripeness. From this analysis, and comparing the values with the mean nevus area (yellow line in Figure 11b), maturation stages from stage 5 are in the same area range as human nevi. This result again agrees with that previously obtained from the qualitative comparison, establishing that certain high maturation stages could be of interest in defining the phantom.

Several diagnostic techniques of human pigmented skin lesions rely on colorimetric characteristic analysis. In order to evaluate the potential of banana spots as phantoms of pigmented skin lesion for this type of technique, a colorimetric analysis was performed on the images obtained for the samples analyzed with the homemade probe. This analysis also considered the skin phototype of the volunteer (Figure 13), to see if there would be a dependence of this factor in the analysis. Measurements of the RGB colorimetric characteristics of healthy skin were performed at two locations: in the area where the spectral measurements were made and at another location nearby. Table 2 shows that, in general, the differences between the two locations for each volunteer were not significant, although in some specific channels of some volunteers differences of around 18% were observed (see Table 2). With these data, the analysis of the volunteer’s skin phototype is significant. Subsequently, the colorimetric characteristics of the nevi were analyzed. Figure 14 shows images of the shades of human nevi. A cross-comparison was performed between the average RGB values of all nevi and those of all banana spots evaluated with the homemade probe. The results shown in Table 3 and Figure 15 indicate the banana spot with the lowest minimum relative error for each human nevus. Regarding the stages of maturation, all the spots belong to stages 4, 5, and 6. This is again in agreement with previous results. The ARE values between human nevi and banana spots are less than 18%, and in some cases, they are below 5%. When these values are compared with the differences between healthy skin locations of the same volunteers, which could reach up to 18%, banana spots are found to be in the same range. This fact indicates their suitability as optical phantoms of human nevi in colorimetric techniques for the diagnosis of pigmented skin lesions.

The spectral similarity between nevi and spots was quantitatively evaluated using the data collected with both probes used. The results of the evaluation of the potential of diagnostic techniques based on direct spectral features are shown in Table 4 and Figure 16. A cross-comparison of the overall and per-spectral-region mean squared error between each human nevus and all banana spots was conducted. As can be seen in Table 4, the minimum global mean squared error is less than 0.07. An analysis of the maturation stage shows that the values are equal to or higher than stage 4, with a majority at stage 5 in this case. A qualitative visual comparison between the most similar spectra for four cases can be seen in Figure 16, first column. Figure 16a,d show high similarity, with the exception of the “W” shape associated with oxygenated hemoglobin. The cases shown in Figure 16b,c present a larger deviation, but still a high similarity.

This spectral analysis was also performed by calculating the mean squared error in three spectral subregions, called R, G, and B, which are analogous to the previous colorimetric channels. As can be seen in the second column of Figure 16, as well as in the values presented in Table 5, there is a notable degree of variability across different regions, with region B exhibiting the most pronounced differences, followed by region G. The increase of the MSE in channel B can be attributed to the presence of noise (see Figure 16), since the lower part of this spectral subrange corresponds to wavelengths in which the optical source used presents low emission. Low optical emission, together with a reduced quantum efficiency response of the linear CCD array detector of the spectrometer, limits the stability of the spectrum below 400 nm and above 800 nm, so the analysis was focused inside this region. The increase in the G channel may be attributed to the aforementioned difference caused by the absence of the “W” spectral shape in banana spots compared to human skin, which presents oxygenated hemoglobin. The error in the R channel, which covers half of the whole spectrum analyzed, is generally low.

For the MSE values obtained with the spectral data taken with the commercial probe, it is possible to observe that this difference is smaller in the entire spectrum evaluated, as shown in Figure 17 and in the values presented in Table 5. This is due to the fact that the sampling volume is smaller and corresponds exclusively to the pigmented zones, where the characteristics mentioned for hemoglobin and chlorophyll are not appreciable in either the nevi or the spots. It is important to note that the spots evaluated with this probe were selected at stage 5 of maturation; we refer to these as “dim spots” due to their low brown color, being the ones for which the lowest MSE values were obtained. In addition, taking into account the dimensions of the nevi and spots, as well as the geometry of the probe, which focuses only on the pigmented regions, could explain why we obtained more accurate results in those zones. Nevertheless, the data collected by the homemade probe should not be dismissed, although the MSE values obtained are higher compared to the commercial probe, they are still low. Furthermore, this configuration enabled a more detailed observation of the characteristics of healthy skin. These findings reinforce the potential of banana spots as phantoms for pigmented skin lesions in diffuse reflectance spectroscopy techniques.

There are also diagnostic techniques of pigmented skin lesions that do not directly employ spectral information, but preprocessed spectral data. As spectral information is composed of many numerical values, dimensionality reduction techniques, such as principal component analysis, are usually employed. Principal component analysis was applied to spectral information of human nevi and banana spots collected with the commercial probe. Diagnostic techniques usually consider a limited number of principal components. Figure 18 shows the analysis of spectral variance explained as a function of the number of principal components. As 5 components present an explanation of 98.63% of the variance, the analysis was made with 5 and 10 components. In order to compare the information of PCA components of human nevi and banana spots, the Euclidian distance can be calculated. However, this parameter strongly depends on the absolute magnitude of the principal components, apart from the relative difference. In order to avoid this bias, the Mahalanobis distance between PCA components of human nevi and banana spots was calculated, and the results are shown in Table 6, either for five or ten PCA components.

As can be appreciated in Table 6, a couple of banana spots are the most similar to all human nevi according to the values of the PCA components. These spots belong to ripening stages 5 and 6, again in agreement with previous results. The minimum Mahalanobis distance is smaller for five PCA components, below seven, than for ten PCA components, where it can reach almost eleven. This fact indicates that banana spots are more similar to human nevi when using a smaller number of PCA components. This can be explained by considering that slight spectral differences between nevi and spots are probably explained by higher PCA components, and as a consequence their consideration worsens the comparison. Diagnostic techniques tend to use as low as possible a number of PCA components, so as to facilitate postprocessing. Figure 18 shows a comparison of whole spectral information in two cases, from the PCA analysis point of view. Although this qualitative comparison is not very relevant, as the PCA components are in another dimensionality system, the similarity can be appreciated. This spectral similarity is below that observed in Figure 16, where the direct spectral information was compared, as expected. Again, the results show the potential of banana spots as optical phantoms of human nevi in diagnostic techniques that employ PCA analysis.

Previous research works have simulated the sampling volume of optical fiber probes used in biomedical optics when the scattering medium, the human skin, is simulated by multiple-layer optical models. For example, Meglinski and Matcher [67] report that a two-fiber probe separated by a source–detector distance of 250 μm has a very shallow detection depth of around 100 μm in the case of a seven-layer optical model with typical optical properties taken from the literature. Their model also predicts that the higher the spacing between the source fiber (core of 200 μm diameter) and the detector fiber (core of 50 μm diameter), the higher the sampled depth of skin. However, these studies have not focused on modeling the dependence of the spatial detector depth sensitivity (Q(x,z) = < l(r_m_) >) or the sampling volume, SV, as a function of the wavelength. In this study, we adopted the simulation procedure of the above-mentioned authors for modeling the SV of two-fiber fiber-optic probes used in the spectral measurements of this research. The simulation tool, which is freely available at the website https://www.biophotonics.ac.nz/ (accessed on 1 July 2024), and the methodology used for our study, were briefly described in Section 2.7. We used a simplified optical model for the skin including two layers, epidermis and dermis, with thicknesses of 60 μm and 5000 μm, respectively. We found that, essentially, in both simulation cases, for the fiber-optic probes with the short (240 μm) and large (2500 μm) source–detector distances, the predicted sampling volume was wavelength-dependent in the spectral region explored, although for λ = 542 nm and 578 nm this quantity was very similar in each fiber probe. The maximum sampling depth, Z^0^_Max_, was around 450 μm and 1800 μm for the homemade and commercial probe, respectively. In addition to observing local minima in the sampling depth at 542 and 578 nm, which coincide with the maximum absorption peaks in the visible spectrum of hemoglobin, according to Feng et al. [33], in the high-absorption regime, the maximum probing depth, Z _o_^MAX^, decreases as the absorption coefficient, µ_a_, increases, following the behavior Z _o_^MAX^ ~ (µ_a_)^−1/4^. Finally, the sampling volume simulated for the short-distance probe (the commercial one) was always less than the sampling volume simulated for the long-distance probe (the homemade probe) for the simulations performed at all the wavelengths selected (400, 420, 500, 542, 578, 600, 650, 700, 750 nm) in this study. Our findings in this Monte Carlo simulation of the sampling volume are in good agreement with the previously documented studies of sampling volume of Meglisnki and Matcher [34,68] for a seven-layered optical model of human skin, although their simulations were performed on optical properties of the scattering medium for only one wavelength (λ = 633 nm). Moreover, the spectral simulations performed in our research for the two-layered optical model provide adequate information as guidance for selecting and/or designing more appropriate commercial or homemade fiber-optic probe designs that ensure better spectral matching between measured diffuse reflectance spectra of nevi in human skin and brown spots in banana peel. Therefore, the simulation study, based on the Monte Carlo method [41], on tracing optical paths of light within the simplified two-layered optical model of human skin here proposed, may be considered as the first step for easily addressing the essential behavior of the spectral dependence of the sampling volume. So, our study provides a reliable theoretical framework for optimizing future studies probing specific turbid media by diffuse reflectance spectroscopy. To the best of our knowledge, this is the first study where the spectral similarity between melanin-pigmented brown spots in bananas and nevi in skin has been investigated, and we have used a spectral MC simulation of the sampling volume to explain the experimental results obtained. The spectral simulation technique and the optical model of skin used could be applied to a wide range of simulation applications for photon transport in biomedical optics and other related research fields. However, it is worth mentioning that the simulation of a two-layer skin model has certain limitations. Although a multilayered model would be more complete, the two-layered skin model has proven to be of practical use in solving skin optical property extraction problems [29,44].

## 5. Conclusions

The results obtained in this study demonstrated a significant degree of area, colorimetric, spectral, and PCA analysis similarity between the brown spots on banana skin and pigmented lesions in human skin. This similarity was shown to be especially relevant for spots at high ripening stages and with areas similar to nevi, generating better results of the average relative percentage error (ARE) (less than 10%). The differences could be explained as a function of the presence of chlorophylls in banana skin being responsible for a low reflectance in certain wavelength intervals, and the spectral “W” shape associated with oxygenated hemoglobin in human skin when the homemade probe was used. It has been observed that the spot formed on the skin of the banana fruit slightly modifies its tonality during its growth, increasing its area with the ripening stage, reaching areas close to those calculated for the nevi.

The use of banana spots as optical phantoms requires ripening stages 5, 6, or 7 for most cases. The similarity in direct spectral and PCA analysis is quite significant, with a mean squared error (MSE) of 0.02 and Mahalanobis distance of 3.63. This was observed in measurements conducted with a homemade probe. In contrast, the MSE values obtained for the measurements with the commercial probe were as small as 0.0010. The small values found were due to the probe operating within the pigmented region of interest. One potential limitation of this study is that despite the presence of tyrosinase in both banana spots [19] and melanoma lesions [14,15,16,17,18], our samples in this study do not contain malignant melanin pigmentation that could slightly modify the spectral similarity obtained in our current study. This issue could be the subject of future research to shed more light on our current results.

The objective of the spectral simulation of the sampling volume was to provide quantitative values of the maximum probing depth, which serve as a basis for explaining the degree of similarity between the experimentally measured diffuse reflectance spectra of banana spots and skin lesions when two types of probes are used in the measurement. A quantitative and qualitative analysis of the results obtained leads to the conclusion that the commercial probe, which has a shorter distance between the detector fiber and the source fiber, results in a significantly lower probing depth than the homemade fiber across the entire spectral region investigated. This may be a factor that could explain the higher similarity between the spectra of the aforementioned zones when using such a commercial measurement probe in comparison to the homemade probe. Some variations were noted between the spectra obtained with the homemade probe, especially before data normalization, for the spectral response obtained for volunteers classified as skin phototypes I and V, given the different levels of pigmentation. These differences were significantly smaller with the use of the commercial probe. Based on these findings, and the limitations of the simulation model, future studies should address a more detailed spectral simulation of the sampling volume using a more complex optical skin model, with a larger number of layers and taking into account the variability in the physiological parameters in each layer.

The results presented in this work show the potential interest of employing banana spots as pigmented lesion phantoms, either for a colorimetric, purely spectral, or PCA analysis. As long as the ripening stage is between 5 and 7, it is possible to select appropriate banana spots with adequate areas and use them as inexpensive and harmless phantoms in the design of optical medical devices for skin pigmented lesion diagnosis.

## Figures and Tables

**Table 1:** Values of the physiological and scattering parameters that are used to define the absorption µ_a_ (λ) and scattering µ_s_ (λ) coefficients.

Layer	Layer Name	B	S	W	F	M	µ_s.500nm_^’^	f_Rayleigh_	bMie	g
1	EPIDERMIS	0	0.90	0.75	0	0.03	40.0	0.0	1.0	0.9
2	DERMIS	0.002	0.90	0.65	0	0	42.5	0.62	1.0	0.9

**Table 2:** Average value of the color intensity in two regions of healthy skin for each volunteer: the first one at the area measured with DRS, and the second at a healthy skin region not measured with DRS (separated by a slash). The relative percentage error (Er(%)) was calculated considering the region not measured with DRS as the reference value.

Volunteer	6	3	4	1	2	7	9	8	10	5
Phototype	I	II	II	III	III	III	III	IV	IV	V
Channel R	203/226	195/207	180/185	225/213	195/233	196/185	214/215	204/172	188/192	218/200
Er(%)	10.17%	5.79%	2.70%	5.60%	16.30%	5.94%	0.46%	18.60%	2.08%	9%
Channel G	128/147	141/139	120/124	153/141	127/157	138/127	139/141	124/108	139/149	141/135
Er(%)	12.92%	1.43%	3.22%	8.51%	19.10%	8.66%	1.41%	14.81%	6.71%	4.44%
Channel B	96/108	110/108	87/90	108/98	89/109	102/92	105/108	94/87	120/129	102/95
Er(%)	11.11%	1.85%	3.33%	10.20%	18.34%	10.86%	2.77%	8.04%	6.97%	7.36%

**Table 3:** Minimum average relative error (ARE) obtained for each nevus after calculating and averaging the Er(%)_N_ of each RGB channel of each nevus–spot combination.

Nevus	Phototype	Spot (Sp)	Stage (S)	ARE
N1	III	29	6	13.02
N2	III	29	6	6.76
N3	II	1	4	10.78
N4	II	1	4	4.51
N5	V	1	4	6.66
N6	I	29	6	17.53
N7	III	2	5	7.93
N8	IV	29	6	14.40
N9	III	29	6	17.43
N10	IV	29	6	4.85

Note: In order to facilitate the discussion of the results, the spots were numbered from Sp1 to Sp48 according to the time of their measurement, and also classified with the corresponding stage of maturation of the sample (from S1 to S7).

**Table 4:** Minimum mean squared error for the spectral comparison between nevi and spots obtained for the homemade probe over the whole spectrum, and for each RGB channel.

Overall Mean Squared Error (400–750 nm)				Mean Squared Error for Each Spectral Region								
Nevus (N) Volunteer	Spot (Sp)	Stage (S)	MSE	Region (R) (571–750 nm)			Region (G) (501–570 nm)			Region (B) (400–500 nm)		
				Spot (Sp)	Stage (S)	MSE	Spot (Sp)	Stage (S)	MSE	Spot (Sp)	Stage (S)	MSE
N1	23	5	0.02	22	4	0.04	23	5	0.06	27	4	0.26
N2	9	5	0.04	29	6	0.05	23	5	0.13	27	4	0.39
N3	23	5	0.07	29	6	0.17	23	5	1.42	27	4	0.34
N4	41	7	0.05	22	4	0.01	41	7	0.22	28	6	1.08
N5	23	5	0.03	29	6	0.02	41	7	0.14	24	6	0.45
N6	22	4	0.07	27	4	0.19	41	7	0.92	20	3	0.58
N7	23	5	0.03	21	4	0.05	41	7	0.08	18	1	0.24
N8	23	5	0.05	29	6	0.01	41	7	0.32	18	1	0.57
N9	23	5	0.04	22	4	0.06	41	7	0.33	18	1	0.08
N10	23	5	0.06	29	6	0.13	23	5	0.33	27	4	0.33

**Table 5:** Minimum mean squared error for the spectral comparison between nevi and spots obtained for the commercial probe in the whole spectrum (400–750 nm).

Overall Mean Squared Error (400–750 nm)			
Nevus (N) Volunteer	Spot (Sp)	Stage (S)	MSE
N1	18	5	0.0086
N2	23	6	0.0011
N3	14	5	0.0040
N4	22	5	0.0010
N5	18	5	0.0056
N6	18	5	0.0049
N7	18	5	0.0087
N8-1	3	6	0.0045
N8-2	7	6	0.0017
N8-3	24	6	0.0037

**Table 6:** Minimum Mahalanobis distance obtained for comparisons between nevi and spots for 5 PCA components (98.63% variance), and with 10 PCA components (99.53%).

	5 PCA Components			10 PCA Components		
Nevus	Minimum Distance	Spot (Sp)	Stage (S)	Minimum Distance	Spot (Sp)	Stage (S)
1	4.3963	24	6	7.5821	23	5
2	4.7922	24	6	8.1107	23	5
3	5.6504	24	6	9.5407	23	5
4	5.4813	23	5	10.0970	23	5
5	3.8875	24	6	8.7439	23	5
6	6.8462	24	6	10.3077	23	5
7	3.6303	24	6	10.2717	23	5
8	6.5337	24	6	10.1623	23	5
9	5.6986	24	6	10.8603	23	5
10	4.3554	24	6	9.6948	23	5

## Data Availability

Data available on reasonable request to the authors.
